# Bioflocculants Produced by Bacterial Strains Isolated from Palm Oil Mill Effluent for Application in the Removal of Eriochrome Black T Dye from Water

**DOI:** 10.3390/polym12071545

**Published:** 2020-07-13

**Authors:** Syed Zaghum Abbas, Yang-Chun Yong, Moonis Ali Khan, Masoom Raza Siddiqui, Afnan Ali Hussain Hakami, Shareefa Ahmed Alshareef, Marta Otero, Mohd Rafatullah

**Affiliations:** 1 Biofuels Institute, School of Environment, Jiangsu University, Zhenjiang 212013, China; Zaghum2009@yahoo.com (S.Z.A.); ycyong@ujs.edu.cn (Y.-C.Y.); 2 Chemistry Department, College of Science, King Saud University, Riyadh 11451, Saudi Arabia; mokhan@ksu.edu.sa (M.A.K.); mrsiddiqui@ksu.edu.sa (M.R.S.); 437203979@student.ksu.edu.sa (A.A.H.H.); 438203872@student.ksu.edu.sa (S.A.A.); 3 CESAM—Centre for Environmental and Marine Studies, Department of Environment and Planning, University of Aveiro, Campus de Santiago, 3810-193 Aveiro, Portugal; 4 Division of Environmental Technology, School of Industrial Technology, Universiti Sains Malaysia, Penang 11800, Malaysia

**Keywords:** bioflocculants, biopolymers, cations, synthetic dyes, wastewater treatment

## Abstract

Four strains of bioflocculant-producing bacteria were isolated from a palm oil mill effluent (POME). The four bacterial strains were identified as *Pseudomonas alcaliphila* (B1), *Pseudomonas oleovorans* (B2), *Pseudomonas chengduensis* (B3), and *Bacillus nitratireducens* (B4) by molecular identification. Among the four bacterial strains, *Bacillus nitratireducens* (B4) achieved the highest flocculating activity (49.15%) towards kaolin clay suspension after eight hours of cultivation time and was selected for further studies. The optimum conditions for Eriochrome Black T (EBT) flocculation regarding initial pH, type of cation, and B4 dosage were determined to be pH 2, Ca^2^⁺ cations, and a dosage of 250 mL/L of nutrient broth containing B4. Under these conditions, above 90% of EBT dye removal was attained. Fourier transform infrared spectroscopic (FT-IR) analysis of the bioflocculant revealed the presence of hydroxyl, alkyl, carboxyl, and amino groups. This bioflocculant was demonstrated to possess a good flocculating activity, being a promissory, low-cost, harmless, and environmentally friendly alternative for the treatment of effluents contaminated with dyes.

## 1. Introduction

Dyes are chemical compounds that show affinity towards the applied substrate, such as surfaces or fabrics to impart color. Synthetic dyes are widely used in numerous industries, for example, in cosmetics, textile, paper, leather tanning, food processing, plastic, rubber, printing, and dye manufacturing [1,2]. Certain kinds of dyes can be toxic, carcinogenic, teratogenic, or mutagenic to different microorganisms, animals, and even humans; are persistent environmental pollutants; and may cross entire food chains, providing biomagnification. The presence of dyes from the textile industry in the aquatic environment is greatly noticeable and unpleasant, even at low concentrations. Conventional wastewater treatments do not guarantee complete removal of dyes due to factors, such as color fastness, stability, and resistance to degradation. In addition, although physicochemical treatments are applied in industries to eliminate dyes from the wastewater, these treatment methods are normally costly and require huge infrastructure on a big piece of land. Even if these treatments improve the removal of dyes, the associated production of concentrated sludge will create another problem related with proper transportation and disposal [3,4]. Therefore, more advanced and improved methods are needed in order to enhance the removal efficiency of these carcinogenic dyes from wastewater [5,6,7].

Coagulation/flocculation processes are amongst the most efficient treatments in the removal of dyes from industrial wastewaters. Flocculants used in wastewater treatment may be divided into three main groups: (a) synthetic organic flocculants (polyacrylamide and polyacrylic acid derivatives); (b) synthetic inorganic flocculants (polyaluminum chloride and aluminum sulfate); (c) bioflocculants [8,9]. The first two groups are broadly employed industrially due to their effectiveness and low-cost; however, the main issue is that they are harmful to human health and are not eco-friendly [10]. For example, acrylamide monomers of polyacrylamide have been found to be both carcinogenic and neurotoxic to humans [11]. It has also been stated that aluminum, the main constituent of polyaluminum, may lead to the development of Alzheimer’s disease [12,13]. Consequently, searching for an alternative, environmentally friendly, and safer substitutes to these chemical flocculants has been the focus of recent research [14,15,16].

Bioflocculants are becoming more popular over the last few years after attracting significant attention in the scientific and biotechnological fields because they are low-cost, have higher efficiency and greater biodegradability compared to conventional flocculants [17]. This is because bioflocculants provide many advantages in terms of cost-effectiveness, safety to human health, easy biodegradability, and free of secondary pollution risks [18]. However, the implementation of bioflocculants in the industries wastewater treatment plants is being hampered due to the complexity and challenges in controlling the bioflocculation, whose practicality and efficiency is affected by factors, such as species, pH, and nutrient conditions [19]. In this context, the present study aimed at the assessment of the removal of dyes by bioflocculants produced by bacterial strains isolated from palm oil mill effluent (POME). For such a purpose, using Eriochrome Black T (EBT) as a model dye, bioflocculant-producing bacterial strains were isolated and tested for their removal from water to select the most efficient one. The selected strain was used for the optimization of initial pH, cation, and bioflocculant dosage to achieve maximum flocculating activity towards the removal of EBT from the solution.

## 2. Materials and Methods

### 2.1. A Sampling of Palm Oil Mill Effluent (POME)

Palm oil mill effluent was obtained from the anaerobic wastewater pond of United Oil Palm Industries Sdn. Bhd., which is situated in Nibong Tebal, Pulau Pinang, Malaysia. The wastewater samples were stored in sterilized high-density polyethylene (HDPE) sampling bottles, stored in an icebox at 4 °C during transportation, and kept at 4 °C until use. The physicochemical properties of raw POME are given in Table 1.

### 2.2. Isolation of Bioflocculant-Producing Bacteria

Serial dilution was carried out by adding 1 mL of raw POME to 9 mL of sterile saline water with a concentration of 9 g/L to obtain a 1:10 dilution. The dilution was continued until a dilution of 1:100,000 was achieved. Then, 0.1 mL of the diluted POME sample was pipetted onto the surface of nutrient agar. After spreading on the agar plates, the plates were placed in an incubator at 37.5 °C overnight in an inverted position. Pure bacterial colonies were obtained by streaking repetition, and isolated colonies were named as B1, B2, B3, and B4. The isolated colonies were then inoculated in four different 20 mL storage bottles that contained 15 mL of a nutrient broth consisting of 5 g/L peptone from meat and 3 g/L meat extract, the broth being autoclaved at 121 °C for 15 min before usage. After incubation overnight at 37.5 °C under shaking at 180 rpm, the four storage bottles, which acted as the stock culture, were stored at 4 °C for further research.

### 2.3. Morphological and Molecular Identification of Bioflocculant-Producing Bacteria

Gram staining was performed prior to the examination of the bacterial strains under a light microscope (Nikon Eclipse 50i, Medtronic, Dublin Ireland). The morphology of the four bioflocculant-producing bacteria, namely, B1, B2, B3, and B4, was observed and determined using a scanning electron microscope (SEM) (FEI Quanta 650 FEG, Tokyo, Japan).

For molecular identification, 12 Petri plates containing three replicates of each of the four bioflocculant-producing bacteria were used for the determination of the 16S rRNA gene sequences, which were compared with the database of the National Center for Biotechnology Information (NCBI) (Pulau Pinang, Malaysia).

### 2.4. Flocculating Activity of Bioflocculant-Producing Bacteria in Kaolin Clay Suspension

The flocculating activity of bioflocculant-producing bacteria was determined according to the method by Kurane et al. [20] with some modifications. Briefly, 180 mL of kaolin clay suspension (5 g/L), 2 mL of culture broth containing the bacterial strain, and 20 mL of 10 g/L calcium chloride (CaCl_2_) solution were added sequentially in a Jar tester (VELP Scientifica JLT 6, Uckfield, United Kingdom), rapidly mixed (300 rpm) during three minutes, then slowly mixed (100 rpm) for ten minutes, and finally left to settle for five minutes. Next, 10 mL of the supernatant was withdrawn using an automatic pipette (Eppendorf Research Plus, Singapore) and used to measure the optical density (OD) at 550 nm (OD_550_) by using a spectrophotometer (HACH DR 2800). Similarly, a blank control without the addition of culture broth was prepared, and its OD_550_ was measured. The experiment was repeated every 2 h and stopped at the end of 12 h. The flocculating activity (%) of the bioflocculant-producing bacteria was calculated using Equation (1). The bioflocculant-producing bacteria that demonstrated the highest flocculating rate were selected for further studies.

(1) $$Flocculating\;Activity\;\left(\%\right)=\frac{A_{0}-A}{A_{0}}\times 100$$

where *A* represents the sample’s OD_550_, and *A_0_* represents the control’s OD_550_.

### 2.5. Effect of Initial pH, Cations, and Dosage on the Flocculating Activity of Bioflocculant Bacteria towards Eriochrome Black T

The removal of EBT from dye solution by the selected bioflocculant-producing bacteria was assessed, and the optimum conditions in terms of initial pH, type of cations, and bioflocculant dosage were determined. For all experiments, the dye solution was prepared by mixing 10 mL of EBT (1 g/L) with 390 mL of deionized (D.I) water. All mixtures were stirred at 120 rpm for two minutes in the Jar test, followed by stirring at 30 rpm for 30 min and then left to settle for 30 min. After settling, the supernatants were collected for OD measurement at 485 nm (OD_485_) by using a spectrophotometer. Experiments were carried out at varied initial pH values, namely, 2, 3, 4, 6, and 8, which were adjusted using NaOH (1 M) and H_2_SO_4_ (0.5 M). After adjusting pH to the corresponding values, 100 mL of nutrient broth, containing the selected bioflocculant-producing bacteria, and 5 mL of 10 g/L CaCl_2_ solution were added to the EBT solution. The described procedure was also carried out for the blank controls at the different pH values, but without the addition of any nutrient broth.

The most favorable cation for the bioflocculant to achieve maximum efficiency in the removal of EBT was determined by using different types of inorganic salts, namely, potassium chloride (KCl), aluminum chloride (AlCl_3_)_,_ and calcium chloride (CaCl_2_), and magnesium chloride (MgCl_2_) at a concentration of 10 g/L. The 100 mL of nutrient broth, containing the selected bioflocculant-producing bacteria, and 5 mL of inorganic salt solution were added to the EBT solution at the optimum initial pH (2). The procedure was repeated for the considered inorganic salts and also for the corresponding blank controls without the addition of any nutrient broth.

The necessary bioflocculant dosage for maximizing the dye removal efficiency was determined by testing different volumes of nutrient broth (50, 75, 100, 125, and 150 mL) of the selected bioflocculant-producing bacteria and 5 mL of each of the considered inorganic salt solutions (KCl, AlCl_3,_ CaCl_2_, and MgCl_2_, 10 g/L) to the EBT solution. The procedure was also carried out with just nutrient broth containing the selected bioflocculant-producing bacteria without the addition of inorganic salts and also for the blank control without the addition of any nutrient broth.

### 2.6. Bioflocculant Purification and Characterization

The bioflocculant produced by the selected bacterial strain underwent purification according to the method by Bar-Or and Shilo and Salehizadeh, et al. with minor modifications. Briefly, the culture broth containing the bioflocculant-producing bacteria was centrifuged at 5000 rpm for 30 min [21,22]. Then, the supernatant was added with two volumes of cold ethanol and left at 4 °C overnight. After centrifugation at 5000 × g for 30 min, the precipitate was collected, and the crude bioflocculant was obtained. The spectrum for the crude bioflocculant was obtained using a Fourier transform infrared spectrophotometer (FT-IR) (Shimadzu IRPrestige-21, Kyoto, Japan), and the functional groups were identified.

## 3. Results and Discussion

### 3.1. Morphological and Molecular Characterization of Bioflocculant Bacteria

**Figure 1:**
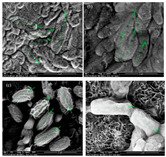
(**a**) SEM image of bacterial strain B1 at 60,000× magnification; (**b**) SEM image of bacterial strain B2 at 60,000× magnification; (**c**) SEM image of bacterial strain B3 at 60,000× magnification; (**d**) SEM image of bacterial strain B4 at 60,000× magnification.

The morphologies of the four bioflocculant-producing bacteria, namely, B1, B2, B3, and B4, were determined after observing their colonies and analyzing the images captured by scanning electron microscope (SEM), as shown in Figure 1. The colonies of bacterial strains B1, B2, and B3 were off-white, non-transparent, and circular, whereas the colonies of bacterial strain B4 were off-white, non-transparent, and rhizoidal after incubation at 37.5 °C for 24 h on nutrient agar. All cells of bacterial strains B1, B2, B3, and B4 had a rough surface and were Gram-negative and rod-shaped. The width/length for the cells of bacterial strains B1, B2, B3, and B4 was 0.3–0.5/1.0–2.4 µm, 0.4–0.8/1.3–1.8 µm, 0.5–1.0/1.3–1.7 µm, and 1.0–1.2/1.9–2.6 µm, respectively.

The NCBI BLAST search of the 16S rRNA gene against GenBank showed that bacterial strain B1 was 99% similar to *Pseudomonas alcaliphila* strain NBRC 102411 (Accession number NR_114072.1); strain B2 was 99% similar to *Pseudomonas oleovorans subsp. lubricantis* strain RS1 (Accession number NR_115874.1); strain B3 was 99% similar to *Pseudomonas chengduensis* strain MBR (Accession number NR_125523.1); strain B4 was 99% similar to *Bacillus nitratireducens* strain 4049 (Accession number NR_157732.1).

### 3.2. Flocculating Activity of Bioflocculant-Producing Bacteria in Kaolin Clay Suspension

**Figure 2:**
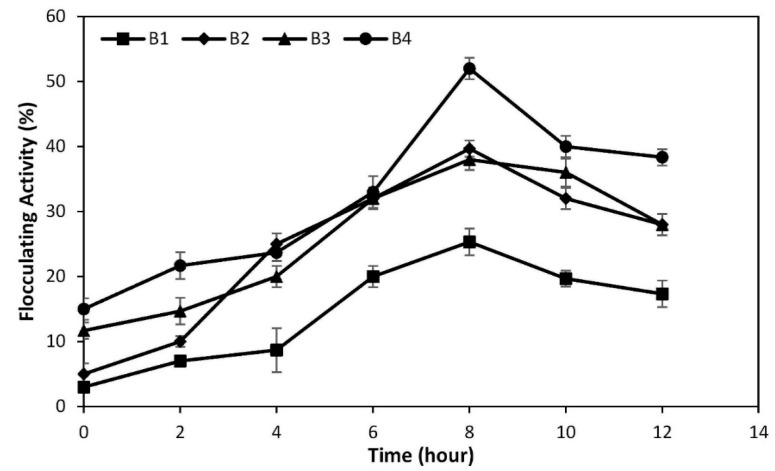
The flocculating activity of bioflocculant from *Pseudomonas alcaliphila* (B1), *Pseudomonas oleovorans subsp. Lubricantis* (B2), *Pseudomonas chengduensis* (B3), and *Bacillus nitratireducens* (B4) towards kaolin clay suspension. Experiment composition: 180 mL of kaolin suspension (5 g/L), 2 mL of culture broth containing the corresponding bacterial strain (B1, B2, B3, or B4), and 20 mL of CaCl_2_ solution (10 g/L).

The flocculating activity throughout time by the bioflocculants from *Pseudomonas alcaliphila* (B1), *Pseudomonas oleovorans subsp. lubricantis* (B2), *Pseudomonas chengduensis* (B3), and *Bacillus nitratireducens* (B4) in kaolin clay suspension is shown in Figure 2. As it might be seen, the four strains showed an increase of flocculating activity with time until reaching a maximum after 8 h of cultivation, and then a decrease in activity was observed. Bioflocculant produced by *Bacillus nitratireducens* (B4) achieved the highest flocculating activity towards kaolin clay suspension among the four bacterial strains, with a maximum flocculating activity of 49.15% under the considered experimental conditions. Meanwhile, bioflocculant from *Pseudomonas alcaliphila* (B1) recorded the lowest flocculating activity with a maximum flocculating activity of 26.47% after 8 h of cultivation. Therefore, *Bacillus nitratireducens* (B4) was a selected bioflocculant-producing strain for further analysis in this research study.

### 3.3. Effect of Initial pH on the Flocculating Activity of Bacillus Nitratireducens towards Eriochrome Black T Synthetic Dye Wastewater

**Figure 3:**
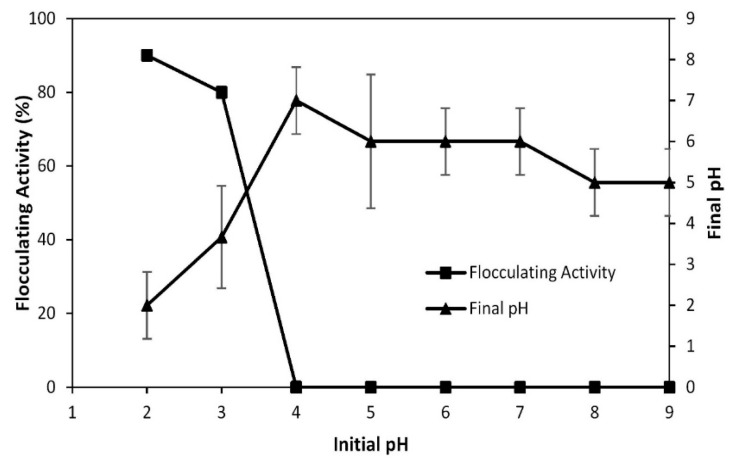
Effect of initial pH on the flocculating activity of bioflocculant from *Bacillus nitratireducens* towards Eriochrome Black T (EBT) solution. Experiment composition: 10 mL of EBT (1 g/L) with 390 mL of deionized (D.I), pH adjusted with NaOH (1 M) and/or H_2_SO_4_ (0.5 M), 100 mL of nutrient broth containing *Bacillus nitratireducens,* and 5 mL of CaCl_2_ solution (10 g/L).

The effect of initial pH on the flocculating activity of bioflocculant from *Bacillus nitratireducens* towards EBT solution was studied within 2 and 8 h, and the obtained results are shown in Figure 3. The maximum flocculating activity of bioflocculant from *Bacillus nitratireducens* towards EBT solution was 86.25%, which was achieved at an initial pH 2. The flocculating activity decreased with increasing initial pH, and the minimum flocculating activity recorded was 0.14% at initial pH 6. The research carried out by Yokoi et al. also reported that bioflocculant from *Bacillus* sp. PY-90 achieved a high rate of flocculation at acidic pH, namely, within the range of 3–5 [23]. Similarly, the findings from Yokoi et al. on bioflocculant from *Enterobacter* sp. BY-29 showed maximum flocculating activity at pH 3, with a decrease with the increase in the pH. Likewise, research by Shimofuruya et al. reported that bioflocculant from *Streptomyces griseus* showed high flocculating activity at the pH range of 2–6, with a maximum at pH 4 [24,25]. This behavior may be related to the effect of solution pH on the aqueous chemistry and on the surface charge of bioflocculants [26]. At acidic pH, the higher H⁺ ions concentration increases the relative concentration of the positively charged groups on the bioflocculant surface, which has been observed to enhance the decolorization of negatively charged dyes, such as EBT [26]. Indeed, it has been already observed for EBT that lowering pH results in higher dye flocculation, as the adsorption of the anionic dye on the positively charged surface of the biomaterial is favored [27].

### 3.4. Effect of Cations on the Flocculating Activity of Bacillus Nitratireducens towards Eriochrome Black T Solution

**Figure 4:**
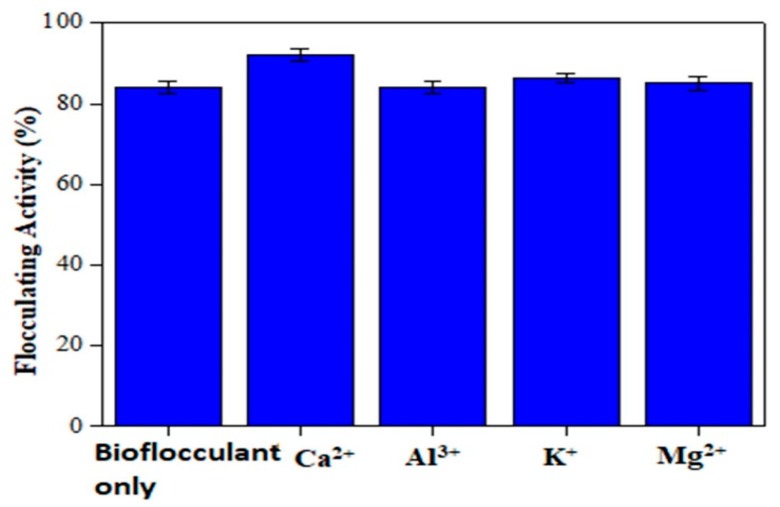
The effect of cations concentration on the flocculating activity of bioflocculant dosage from *Bacillus nitratireducens* towards EBT solution. Experiment composition: 10 mL of EBT (1 g/L) with 390 mL of deionized (D.I), pH 2 adjusted with NaOH (1 M) and/or H_2_SO_4_ (0.5 M), 100 mL of nutrient broth containing *Bacillus nitratireducens,* and 5 mL of salt solution (CaCl_2_, AlCl_3_, KCl, or MgCl_2_) at a concentration of 10 g/L (except for the experiments with only bioflocculant, with no salt).

Cations are usually introduced to wastewater to increase the flocculating activity of the bioflocculants [13]. The effect of cations on the flocculating activity of bioflocculant from *Bacillus nitratireducens* towards the EBT solution is shown in Figure 4. As it might be seen, all cations successfully increased the flocculating activity of the bioflocculant from *Bacillus nitratireducens* to above 87%. The flocculating activity of the bioflocculant without the addition of any cation to the wastewater was just 85.48%. With the addition of cations, a maximum flocculating activity of 89.63% was achieved with Ca^2+^, and a minimum flocculating activity of 87.14% was obtained when Al^3+^ was added to the wastewater. Similar findings from Yokoi et al. showed that the addition of divalent cations enhanced the rate of flocculation of poly-γ-glutamic acid (PGA) produced by *Bacillus* sp. PY-90 towards kaolin [28].

Similar to results in Figure 4, Gong et al. found that divalent metal ions, such as Ca^2^⁺ and Mg^2^⁺, achieved a better rate of flocculation compared to trivalent metal ions, such as Al^3^⁺ and Fe^3^⁺, and also monovalent metal ions, such as Na⁺ and K⁺ [29]. According to these authors, this occurred because divalent metal ions were able to destabilize the negative charges on the particles through neutralization and bridging [29]. Once that bridging between molecules and bioflocculant is successful, a long flocculant chain of molecules is formed. This flocculant chain can then adsorb other chains to form huge flocs, which are able to settle quickly, leading to an increase in the bioflocculant flocculating activity [30].

It is generally accepted that the flocculation induced by bioflocculant can occur by bridging and charge neutralization. Sheng et al. found that the flocculating rate was generally increased when different metal ions, except Mg^2^⁺, were added [31]. Especially, A1^3^⁺ and Fe^3^⁺ enhanced the flocculating rate. There might be two reasons to account for these outcomes. First, adding cations to the kaolin suspension may decrease the negative electrical charge of the particles. Second, through cation bridging, bioflocculant can absorb onto the clay particles more efficiently and flocculate them easily. The difference between this experiment and other investigations was that Ca^2^⁺ had little effect on the flocculating activity when compared with other metal ions.

### 3.5. Effect of Dosage and Cations on the Flocculating Activity of Bacillus Nitratireducens towards Eriochrome Black T Solution

**Figure 5:**
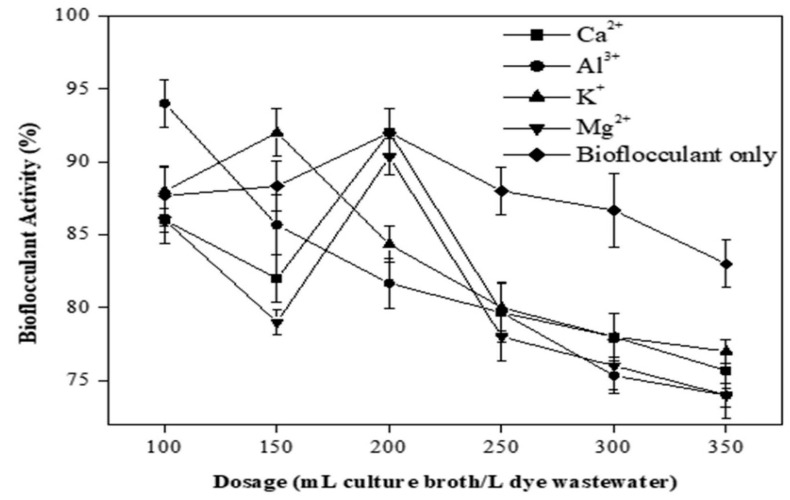
The effect of bioflocculant dosage and cations concentration on the flocculating activity of bioflocculant from *Bacillus nitratireducens* towards EBT solution. Experiments composition: Experiments composition: 10 mL of EBT (1 g/L) with 390 mL of deionized (D.I), pH 2 adjusted with NaOH (1 M) and/or H_2_SO_4_ (0.5 M), different dosages of nutrient broth containing *Bacillus nitratireducens* (50, 75, 100, 125, or 150 mL), and 5 mL of salt solution (CaCl_2_, AlCl_3_, KCl, or MgCl_2_) at a concentration of 10 g/L (except for the experiments with only bioflocculant, with no salt).

The synergic effect of dosage and cations on the flocculating activity of bioflocculant from *Bacillus nitratireducens* towards the EBT solution is shown in Figure 5.

The best dosage of nutrient broth containing bioflocculant from *Bacillus nitratireducens* towards EBT solution was found to be 250 mL/L with the addition of Ca^2^⁺. However, this dosage of nutrient broth containing the bioflocculant is considered high since the ideal range is between 1 and 150 mL/L, as reported by Deng et al. [30]. Results in Figure 5 showed that the maximum rate of flocculation at the best bioflocculant dosage was 90%. On the other hand, the lowest flocculating activity among all dosages and cations used was 81% at a dosage of 375 mL/L with the addition of K⁺ to the EBT solution. As a general trend, it was observed that flocculating activity decreased with an increasing dosage of bioflocculant. However, a decrease in flocculation was also observed at relatively high broth dosages. In this sense, the flocculating activity of bioflocculant from *Bacillus nitratireducens* started to decrease when the dosage exceeded 125 mL/L with the addition of Al^3^⁺ to the EBT solution. Similar trends were also observed under the addition of K⁺ (broth dosage > 187.5 mL/L), Mg^2^⁺ (broth dosage = 250 mL/L), and Ca^2^⁺ (broth dosage = 250 mL/L). The research by Gong et al. reported that an increase or decrease in bioflocculant concentration over the optimum dosage might cause lower rates of flocculation [29]. When bioflocculant dosage is insufficient, bridging phenomena cannot effectively occur. On the other hand, an excess of bioflocculant may cause competition and repulsion, which interfere with bridging and may also reduce effective volume, which also leads to poor settability. In any case, flocs formation is reduced, and the settling of the particles is more difficult and time-consuming. Liu et al. found that in the range of 0.3–8.2 mg/L, the flocculating rate was over 90%, and at maximum bioflocculant dosage of 1.2 mg/L, the optimum flocculating rate was observed [32]. The poorer flocculating rate was normally achieved at a higher and lower dosage. The bridging phenomena could not successfully occur when bioflocculant dosage was inadequate. The destabilization of kaolin particles could be explained by the change of charge of the solution after the excessive addition of bioflocculants. The excessive addition of negatively charged bioflocculant caused the repulsion of negatively charged kaolin particles and poor stability.

### 3.6. Bioflocculant Characterization of Bacillus Nitratireducens

**Figure 6:**
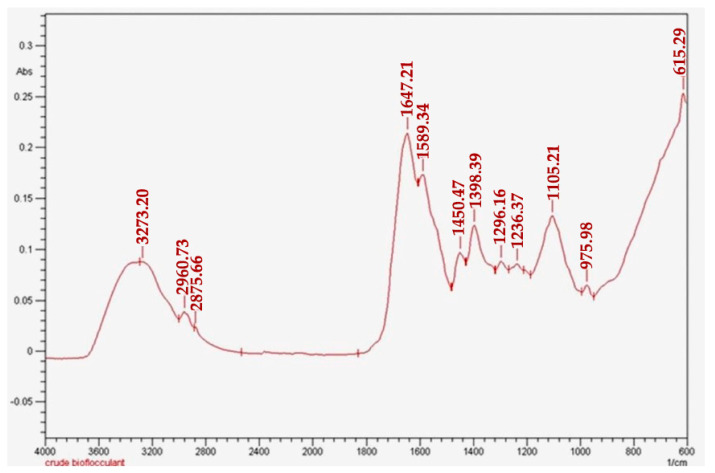
FT-IR spectrum of crude bioflocculant from *Bacillus nitratireducens*.

The FT-IR spectrum obtained for the crude bioflocculant from *Bacillus nitratireducens* is shown in Figure 6.

The functional groups present in the bioflocculant from *Bacillus nitratireducens* (Figure 6) were identified based on the peaks indicated in the spectrum and by referring to the simplified infrared correlation table prepared by Pavia et al. [33]. The first large band with strong intensity at around 1647.21 cm^−1^ showed C=O stretching and was identified as amide I. N–H bending with a medium to the strong intensity at around 1589.34 cm^−1^ was identified as associated with primary and secondary amines and amides. The carboxylic acid functional group with O–H stretching was signified by the band with medium intensity at 3273.20 cm^−1^. Besides, the bands at 1450.47 and 1398.39 cm^−1^ with medium intensity indicated CH_3_ bending. Lastly, the bands at 1105.21 and 975.98 cm^−1^ that fell within the 1200 to 900 cm^−1^ range were caused by C–O bonds in different polysaccharides, as shown in Table 2.

## 4. Conclusions

Four bacterial bioflocculant-producing strains were isolated from POME and identified as *Pseudomonas alcaliphila* (B1), *Pseudomonas oleovorans* (B2), *Pseudomonas chengduensis* (B3), and *Bacillus nitratireducens* (B4). Among these strains, *Bacillus nitratireducens* (B4) showed the highest flocculating activity towards a kaolin clay suspension and was selected for further studies on the removal of EBT dye from water. The effects of initial pH, type of cation, and bioflocculant dosage by *Bacillus nitratireducens* on the treatment of EBT solution were shown to be notorious, and these operation factors were optimized. Maximum flocculation of 90% was attained under initial pH 2, the presence of Ca^2^⁺, and a bioflocculant dosage of 250 mL/L. FT-IR analysis evidenced the presence of hydroxyl, alkyl, carboxyl, and amino groups in the bioflocculant, with these groups playing important roles in flocculation. In short, the bioflocculant from *Bacillus nitratireducens* from POME, which was shown to be efficient in the flocculation of EBT, is a promissory, low-cost, harmless, and environmentally friendly alternative for the treatment of dye-containing effluents.

## Figures and Tables

**Table 1:** Physicochemical properties of the raw palm oil mill effluents (POME) used in this work.

Physicochemical Property	Raw POME
Temperature (°C)	40 ± 1
pH	8 ± 0.16
Conductivity (μs/cm)	130 ± 0.46
Nitrogen (mg/L)	9 ± 0.37
Phosphate (PO_4_^−3^ mg/L)	20.3 ± 0.25
Total suspended solids (mg/L)	188 ± 1
Dissolved oxygen demand (mg/L)	3.1 ± 0.15
Chemical oxygen demand (mg/L)	46,500 ± 3.55
Biochemical oxygen demand (mg/L)	21,000 ± 3.21

**Table 2:** FT-IR band assignment in the spectrum of crude bioflocculant from *Bacillus nitratireducens*.

Frequency (cm^−1^)	Functional Group
1105.21–975.98	C–O bonds
1450.47–1398.39	CH_3_ bending
1589.34	N–H bending
1647.21	C=O stretching, amide
3273.20	Carboxylic acid, O–H stretching
